# Parental Incarceration and Children’s Risk of Violent Victimization: An Examination of Risks and Perpetrators’ Relationship With the Victim in Finnish Total Population Data

**DOI:** 10.1177/08862605261452168

**Published:** 2026-06-05

**Authors:** Ilona Nissinen, Mikko Aaltonen, Joonas Pitkänen, Pekka Martikainen, Karoliina Suonpää, Antti Latvala

**Affiliations:** 1 University of Helsinki, Finland; 2 University of Eastern Finland, Joensuu, Finland; 3 The Max Planck – University of Helsinki Center for Social Inequalities in Population Health (MaxHel Center), Finland; 4 Max Planck Institute for Demographic Research, Rostock, Germany

**Keywords:** parental incarceration, childhood victimization, violence, register data, total population data

## Abstract

Parental incarceration is associated with harmful outcomes in children, but little is known about the relationship between parental incarceration and offspring violent victimization. Using Finnish total population register data (*N* = 1,017,072), we studied the association between parental incarceration and violent victimization in childhood and adolescence and identified perpetrator’s relationship with the victim. We conducted population-level and stratified Cox regression analyses and estimated cumulative incidence from Kaplan-Meier analysis to study the relative and absolute risks of violent victimization. In the population-level analyses, we examined the general risk of violent victimization among children with and without parental incarceration. In the stratified analyses, we studied the risk of violent victimization in relation to the length of co-residence with the parent. We further analyzed relationships between victims and perpetrators. We found that parental incarceration was associated with a notably elevated risk for violent victimization in childhood and adolescence (hazard ratios of 2.4–4.4). The highest relative risks were found for maternal incarceration. The associations between parental incarceration and violent victimization were stronger among children who had co-resided longer with the parent. The absolute risks were the highest for children with incarcerated mothers regardless of co-residence. Among children with parental incarceration, a notable proportion (21.2%–36.4%) of violent victimization was perpetrated by an adult outside the family. Our findings highlight that children with parental incarceration are at a high risk of being violently victimized in childhood and adolescence, and a substantial part of the violence is conducted by someone outside the family. Better identification of violence among children with parental incarceration is needed.

## Introduction

Violent victimization is linked to harmful outcomes in children. In addition to the potential permanent or long-lasting injuries, victimization can also have a cascading effect on other aspects of health: mental health problems (Baldwin et al., 2023), internalizing and externalizing behaviors (Mills et al., 2013; Moylan et al., 2010; Strathearn et al., 2020), substance abuse problems (Norman et al., 2012; Strathearn et al., 2020), and poor physical health (Lee et al., 2024) are associated with victimization in childhood and adolescence. Furthermore, childhood abuse is linked to an increased risk of victimization later in life (McIntyre & Widom, 2011).

Several factors may contribute to an increased risk of victimization at young age. First, in addition to the established association between an individual’s externalizing or violent behavior and related risk of victimization (Jennings et al., 2012), research has identified a link between parental criminality and a heightened risk of offspring victimization (Järvinen et al., 2024) and child maltreatment (Austin, 2016). Furthermore, various parental correlates of crime have been linked to victimization. For instance, lower socioeconomic status is associated with increased risk of childhood violent victimization (Ellonen et al., 2024), and risk of childhood maltreatment has been found to be elevated among children of parents with substance abuse problems (Choenni et al., 2017).

Parental incarceration (PI) is associated with deleterious outcomes in children such as poorer health (Wildeman et al., 2018), criminal behavior (Besemer et al., 2011; Nissinen et al., 2024; Olsen, 2022; Wildeman & Andersen, 2017), lower educational achievement (Anker, 2023), and premature mortality (van de Weijer et al., 2018), but little is known about the relationship between PI and offspring violent victimization. Multiple theoretical perspectives might be invoked to account for this possible association. First, PI could cause negative strain (Agnew, 1992; Merton, 1938) or stigma (Lemert, 1967), which can be harmful for child development and lead to risky behaviors that might predispose to violent victimization (Berg & Felson, 2020). Criminally active parents could also be violent toward the child or expose the child to other violent individuals and environments with heightened risk of victimization. It should also be noted that, especially in the Nordic context, PI is experienced by a highly selected group of children as incarceration is used as a last resort sanction for severe and/or repetitive criminal behavior. Hence, multiple social and behavioral risk factors for violence accumulate in the families of incarcerated individuals (Luk et al., 2023). On this basis, children with PI are a particularly vulnerable population in relation to victimization. Current research on the link between PI and victimization has yielded somewhat mixed results, with one study suggesting that paternal incarceration predicts maternal neglect and physical aggression toward children, indicating a possible detrimental intra-familial spillover effect (Turney, 2014). Conversely, another study linked PI to children’s elevated risk of childhood neglect, verbal abuse and witnessing violence, but not with elevated relative risk of physical victimization (Källström et al., 2019). However, the study was based on a small sample.

Despite the theoretical and empirical relevance of the topic, previous research suffers from several limitations. First, to our knowledge, the risk of violent victimization among offspring with PI has not been studied with total population register data, which may have resulted in biased estimates due to selected samples. Second, in addition to the general risk of violent victimization, the risks should be considered regarding the age at victimization and the contact between parents and children. Children’s exposure to violent adults and peers within and outside the family may vary by age. Younger children are more dependent on their caregivers and there are typically fewer contacts outside the nuclear family than among older children who are more independent and exposed to various environments. Further, previous research has not examined co-residence with the incarcerated parent as a possible moderating factor for the associations. However, on a population level, children whose father does not live with them have been shown to be at heightened risk of becoming victims of paternal lethal violence (Nobes et al., 2019). This result was consistent for both biological fathers and stepfathers and suggests that the possible mechanism explaining this could be the weaker parent-child bond. Thus, the risk of violent victimization may be different depending on the degree of shared living environment with the incarcerated parent.

There is also limited knowledge about victim–perpetrator relationships in victimization at a young age, and previous research has raised concerns about the lack of data to identify violence outside the family (Devries et al., 2018). Studies on victimization in childhood have largely focused on family-based violence and child maltreatment. Studying the risk of violent victimization beyond the nuclear family context is thus needed to broaden the understanding of victim–perpetrator relationships associated with an increased risk of violent victimization. It is particularly important when examining children with PI, as this group is more likely to be exposed to risky environments and other violent individuals than children without PI.

Taken together, current research on violent victimization among children with PI is scarce, even though it is known that children with PI are exposed to various risk factors of victimization. Existing research has also relied on survey-based data with relatively small sample sizes and possibly serious reporting biases in reporting sensitive topics. Furthermore, the victim–perpetrator relationship is particularly understudied in the case of children with PI. To address these notable gaps in existing literature, we used Finnish register data to shed light on the risk of violent victimization among children with PI in a Nordic penal context. The study has the following aims: (1) Study the associations of PI with offspring violent victimization between ages 0 to 17 years, further separated into 0 to 10 years and 11 to 17 years; (2) study whether PI is differently associated with violent victimization depending on the length of co-residence with the parent; and (3) study the relationships between perpetrator and victim separately for those with and without PI. Building on prior research on PI and offspring outcomes, we propose the following hypotheses: (1) PI is associated with an increased risk of violent victimization, (2) the relative risk of violent victimization is higher for those co-residing longer with the parent, and (3) parental violence is more common among children with versus without PI.

## Methods

Using total population registers (TK/2575/07.03.00/2025, THL/5298/14.06.00/2025), we collected data on all children born in Finland from 1987 to 2003, for whom both biological parents could be identified (*N* = 1,017,072). The registers are routinely maintained by different officials, such as the police and the health care providers, and the data are primarily collected for administrative and statistical purposes. Permissions for the secondary use of these data in research is granted by register authorities (Statistics Finland; Findata). All individuals born in Finland between 1987 and 2003 who were residents of Finland at year-end were obtained from Statistics Finland’s registry of permanent residents in Finland on the last day of each year. The register includes information on individuals’ birth month and year, sex, and pseudonymized personal identification codes, which are issued to everyone residing in Finland. Using these personal identifiers, we linked data from various administrative sources including information on, for example, individual’s educational level, medical records related to external causes of injury (violence), and violent offenses suspected by the police. The children’s identification codes were also linked to the respective codes of their parents, allowing accurate identification of parent-child relationships. Follow-up data were available until the end of 2020. In general, there is typically only little missingness in register data. Small amounts of missing values were observed for some parental variables, detailed in the descriptive results.

### Context of Finland

Key points regarding the context of Finland need to be highlighted. In Finland, most prison sentences last less than a year (Prison and Probation Service of Finland, 2024), and in general, Finnish prison population rate is among the lowest in Europe (Aebi et al., 2023). According to the Criminal Code (39/1889) of Finland, the minimum length for fixed-term unconditional incarceration is 14 days. The maximum is 12 years, or 15 years in the case of a joint sentence for several offenses. The most severe sanction is “life imprisonment,” that is imposed without a fixed end date. With life imprisonment, the earliest possible release is after 12 years, or 10 years if the offense was committed before age 21 (Criminal Code, 39/1889), and the average length of life imprisonment sanction has been around 15 years in the 2020s (Prison and Probation Service of Finland, 2024). Furthermore, Finland is a Nordic welfare state characterized by social policies and public services designed to promote economic security and social well-being. However, incarcerated individuals (Rautanen et al., 2024) and their children (Grönqvist et al., 2025) suffer from various adversities in the Nordic context as well. Studying the risk of violent victimization in this high-risk group is important in order to better identify the support and interventions these children might need.

### Measures

PI was defined using data on parental prison sentences, derived from district court decisions (available for years 1977–2020), from when their offspring were under 18 years old. We used the case number to identify individual adjudications and created separate variables for PI (either parent incarcerated; paternal incarceration; maternal incarceration) occurring at any time as a minor (0–17 years).

Offspring's violent victimization was defined using medical register data (The Care Register for Health Care), the Causes of Death Register and police-reported violent victimization. Medical register data contains inpatient (available from early 1970s) and specialized outpatient treatment (available from 1998 onwards) data on the primary and secondary diagnoses and external causes of morbidity, and the register of causes of death includes data on underlying and contributing causes of death. From these data, we identified cases of medical treatment and deaths due to injury purposefully inflicted by another person (Sariaslan et al., 2024) using codes from the International Classification of Diseases (ICD) version 10 (Supplemental Table 1). We excluded the code for sexual assault by bodily force (Y05), but the code for other maltreatment (Y07) could include forms of sexual abuse. While the codes also included some forms of non-contact violence, such as maltreatment and neglect, they were of such severity that they required hospital treatment and were thus defined as violent victimization. We included violent victimization from the year 1996 onwards when the ICD-10 was in use. The police data (for years 1996–2020) includes, for example, an encrypted identification code for both the perpetrator and the victim, the type of suspected crime, and the date of the offense. We identified suspected violent crimes following the main chapters of the Criminal Code of Finland (39/1889). Violent crimes included petty assaults, assaults (including attempts), and aggravated assaults (including attempts and attempted homicides; Supplemental Table 2).

Co-residence in childhood was measured using Statistics Finland’s annual data on dwellings recorded on the last day of the year until 2019. We examined co-residence during childhood, as contact at home is expected to be closer before the teenage years. We computed the number of years the child and their parents had the same dwelling code at the end of each year, until the child turned 12. To study the possible group differences in shorter versus longer co-residence with the child, the variables were further coded as: (1) a maximum of 6 years lived with the parent(s) and (2) over 6 years lived with the parent(s). The variable was dichotomized to obtain two categories of equal length.

As individual-level covariates, we included child’s sex and birth year. Sociodemographic differences in the risk of violent victimization were addressed by adjusting for established correlates of childhood victimization and parental prison sentences: parent’s immigrant background, parent’s age at childbirth and the highest level of education for parents during the follow-up (Supplemental Table 3). Prior research has shown that low socioeconomic status (Ellonen et al., 2024), immigrant background (Lahti et al., 2024), and parent’s young age (Abate et al., 2025) are associated with violent victimization, and the same correlates are found for (parental) incarceration (Aaltonen et al., 2024; Andersen, 2016; Nissinen et al. 2024).

### Analyses

We conducted covariate-adjusted Cox proportional hazards models with child’s age as the time scale to estimate the association between PI and offspring violent victimization at ages 0 to 17. Separate models were run for exposure to incarceration of either parent, to incarceration of father, and to incarceration of mother. PI was considered a risk indicator rather than a direct causal factor for violent victimization, and to maximize power, PI could occur at any time between the child’s ages of 0 to 17 years in the main analyses. The outcome variable was the first occurrence of violent victimization, either in the medical, causes of death, or police register. Thus, in the main analysis, PI did not necessarily precede victimization. However, this sequence was observed in the majority of cases (Supplemental Table 4). Follow-up ended at the occurrence of the first violent victimization, emigration, death, or end of register coverage in the end of 2020, whichever happened first. All models were adjusted for sex and birth year, and parent’s immigrant background, highest education, and parents age at childbirth. Models with either parent’s incarceration as the exposure variable were adjusted for maternal covariates. In addition to comparing the risks in different groups (relative risks), we examined absolute risks of violent victimization by the age of 18, by the length of co-residence, using cumulative incidence from Kaplan-Meier analysis. Joint examination of relative and absolute risks allows for a more nuanced understanding of risk profiles by integrating information on between-group differences with the overall prevalence of the outcome. Exclusive focus on the relative risk measures may downplay situations in which relative differences are small, but the outcome is common, thus leading to sizable absolute differences. Absolute differences are particularly relevant for assessing the population-level burden of violence and may thus be of particular interest.

We then conducted separate models for violent victimization during childhood (0–10 years) and adolescence (11–17 years) separately. New cases of violent victimization were studied in the adolescence model, excluding those with victimization during childhood. Next, we stratified the analysis by the length of co-residence with the parent to study the possible co-residence related heterogeneity in the associations between PI and violent victimization.

Finally, we studied the relationship between the victim and the perpetrator. We used the following hierarchical categories enabled by the data: the perpetrator was the victim’s (1) biological father, (2) biological mother, or (3) sibling, parent’s spouse, or someone else living in the same apartment at the end of the previous calendar year. The perpetrators not related to the victims were classified based on their age difference relative to the victim, thus the offender could be (4) a peer (age difference 5 years or less), (5) a person with a 6 to 15 years’ age difference with the victim, or (6) someone with more than 15 years’ age difference with the victim. Differences between groups were assessed by estimated proportions and their confidence intervals. Analyses were conducted for victimization at ages 0 to 17 years and separately for children 0 to 6 years old to study early childhood victimization. Before school age, there is likely to be less contact with non-family adults, and thus the risk of violent victimization at home versus outside the home could be higher in comparison to older children. Due to the low number of children being both exposed to parental incarceration and being victims of violence in early childhood, we did not analyze maternal and paternal incarceration separately for violent victimization at 0 to 6 years.

#### Additional Analyses

We first conducted separate models for violent victimization defined using medical register data (including causes of death) and police data. Second, as we did not have full data coverage for the earlier cohorts (1987–1995), to test a possible estimation bias, we ran the main analysis including only individuals born between 1996 and 2003. Third, as violence toward offspring can result in PI, we tested whether the results were affected by restricting PI to occur before the first case of violent victimization. Here, we defined exposure to the first PI to occur at 0 to 5 years or 0 to 11 years and analyzed the new cases of victimization occurring after the first PI (i.e., after the child turned 6 or 12, respectively). Fourth, the risk of violent victimization is likely to be higher for children of parents with a history of violent convictions. Thus, to test whether the association is explained by parental violence, we limited the data to include only those parents who did not have any violent convictions when the child was under 18 years old and studied the association between PI and violent victimization within this population. Fifth, separate survival models were estimated, with outcomes defined as becoming a victim of each perpetrator category included in the main analysis. Finally, regardless of the length of incarceration periods, which are on average less than a year in Finland, they still affect the time of co-residence. We conducted two sensitivity analyses in which co-residence was measured before the parent’s first incarceration. In the first model, co-residence was measured at 0 to 5 years and PI at 6 to 17 years. In the second model, co-residence was measured at the same age as in the main model (0–11 years), but PI was measured at ages 12 to 17 years. In neither model, children could have PI periods during the age range for which co-residence was measured.

## Results

Around 13% of the children with either parent incarcerated had been victimized before they turned 18 years (Table 1, Supplemental Table 5). The proportion was higher with maternal incarceration as 20% of children with maternal incarceration were victimized by age 18. These proportions were notably higher than among children with no PI. The number of victims in the medical and the causes of death register was significantly lower than in the police register. Furthermore, most children were linked to only one violent victimization event, and most cases in the police register were assaults (70%), followed by petty assaults (28%; Supplemental Table 6).

**Table 1.** Violent Victimization and Background Variables by Parental Incarceration Among Children Born in Finland in 1987–2003 With Follow-Up for 1996–2020.

	Either parent incarcerated		Paternal incarceration		Maternal incarceration	
Data and variable details	PI (*n* = 18,707)	No PI (*n* = 998,365)	PI (*n* = 17,600)	No PI (*n* = 999,472)	PI (*n* = 1,859)	No PI (*n* = 1,015,213)
ALL DATA SOURCES, Violent victimization %						
At 0–17 years	12.8	3.4	12.4	3.4	19.6	3.5
At 0–10 years	2.8	0.5	2.7	0.5	5.3	0.5
At 11–17 years	10.5	3.0	10.3	3.0	15.9	3.1
MEDICAL REGISTER, Violent victimization %						
At 0–17 years	1.3	0.3	1.2	0.3	2.1	0.4
DEATH REGISTER, Violent victimization %						
At 0–17 years	0.1	0.01	0.1	0.01	0.5	0.01
POLICE REGISTER, Violent victimization %						
At 0–17 years	12.2	3.2	11.9	3.2	18.5	3.4
Birth year, mean [95 % CI]	1,994.4 [1,994.3, 1,994.5]	1,994.8 [1,994.8, 1,994.8]	1,994.4 [1,994.3, 1,994.5]	1,994.8 [1,994.8, 1,994.8]	1,994.7 [1,994.5, 1,994.9]	1,994.8 [1,994.8, 1,994.8]
Child’s sex, %						
Boy	51.0	51.1	50.8	51.1	52.8	51.1
Girl	49.0	48.9	49.2	48.9	47.2	48.9
Parental immigrant background, %						
Parent born in Finland^a^	89.8	93.8	93.0	96.1	94.0	96.2
Parent born elsewhere^a^	10.2	6.2	7.0	3.9	6.0	3.8
Years lived with parent, mean [95% CI]^b^						
At age 0–11 years	10.7 [10.7, 10.7]	11.8 [11.8, 11.8]	3.9 [3.9, 4.0]	10.1 [10.1, 10.1]	6.8 [6.6, 7.0]	11.6 [11.6, 11.6]
Age of parent at child’s birth, mean [95 % CI]^c^	25.3 [25.2, 25.3]	29.1 [29.1, 29.1]	28.9 [28.8, 29.0]	32.0 [32.0, 32.0]	25.9 [25.6, 26.1]	29.5 [29.5, 29.6]
Parent’s highest education, %						
No secondary education^d^	19.5	3.2	52.3	16.7	61.4	9.3
Upper secondary education^d^	60.0	38.1	41.8	47.0	32.2	42.8
Tertiary education or higher^d^	20.5	58.7	5.9	36.3	6.5	48.0

*Note.* PI at 0–17 years; PI = Parental incarceration.

a Information was missing for 846 fathers and 30 mothers. Information for either parent incarcerated is both parents born in Finland or either parent is born elsewhere.

b Information was missing for 212 children. Information for either parent incarcerated is the mean years living with either parent.

c Information for either parent incarcerated is the mean age of the younger parent.

d Information for either parent incarcerated is either parent has the level of education in question.

**Figure 1.**
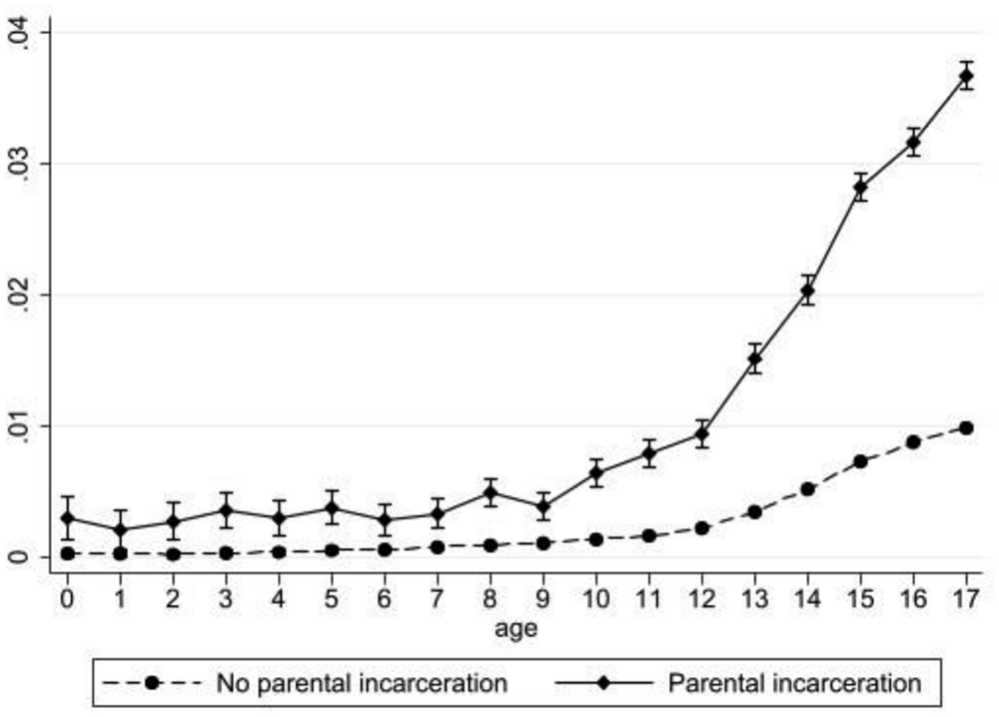
Average number of violent victimization events, with 95% confidence intervals, by age for children with and without parental incarceration among children born in Finland in 1987–2003 with follow-up for 1996–2020.

Children with and without PI demonstrate comparable patterns in average violent victimization events over the course of childhood, with the risk increasing throughout childhood and rising more sharply during the teenage years (Figure 1). Children with PI had more violent victimization events compared to children without PI, with the difference being observed from the first year of life throughout the follow-up. The disparity between these groups widened significantly during adolescence.

In the Cox models (Table 2) using outcomes from all data sources, PI was associated with a notably elevated risk for violent victimization in childhood (HRs between 2.4 and 4.4). The risk was elevated throughout the follow-up, but relative risks were higher in childhood (HRs between 3.6 and 4.4) in comparison to adolescence (HRs between 2.4 and 2.9). Higher relative risks were found for maternal in comparison to paternal incarceration.

**Table 2.** Associations and Stratified Analyses: The Relationship Between Parental Incarceration and Offspring Violent Victimization Among Children Born in Finland in 1987–2003 With Follow-Up for 1996–2020. Stratification was Conducted by the Length of Co-Residence With the Parent. Reference Categories: No Parental/Paternal/Maternal PI.

Violent victimization	Either parent incarcerated				Paternal incarceration				Maternal incarceration			
	HR		[95% CI]		HR		[95% CI]		HR		[95% CI]	
at 0–17 years	2.6		[2.5, 2.7]		2.7		[2.5, 2.8]		3.2		[2.9, 3.6]	
at 0–10 years	3.6		[3.3, 4.0]		3.7		[3.4, 4.1]		4.4		[3.6, 5.4]	
at 11–17 years	2.4		[2.3, 2.6]		2.5		[2.3, 2.6]		2.9		[2.6, 3.3]	
Violent victimization	Either parent incarcerated				Paternal incarceration				Maternal incarceration			
	A maximum of 6 years lived with parent^a^		Over 6 years lived with parent^a^		A maximum of 6 years lived with parent^a^		Over 6 years lived with parent^a^		A maximum of 6 years lived with parent^a^		Over 6 years lived with parent^a^	
	HR	[95 % CI]	HR	[95 % CI]	HR	[95 % CI]	HR	[95 % CI]	HR	[95 % CI]	HR	[95 % CI]
at 0–17 years	1.8	[1.5, 2.1]	2.6	[2.5, 2.8]	1.6	[1.5, 1.7]	3.2	[3.0, 3.6]	1.8	[1.5, 2.1]	4.0	[3.4, 4.6]
at 0–10 years	1.8	[1.4, 2.4]	3.5	[3.2, 3.9]	1.9	[1.7, 2.1]	5.2	[4.2, 6.5]	1.6	[1.1, 2.2]	6.4	[4.9, 8.5]
at 11–17 years	1.8	[1.5, 2.2]	2.5	[2.3, 2.6]	1.6	[1.5, 1.6]	3.0	[2.7, 3.3]	1.9	[1.5, 2.3]	3.5	[2.9, 4.1]

*Note.* Violent victimization was measured using data from the medical, causes of death and police registers. PI at 0–17 years. All models were adjusted for child’s sex, birth year, parental immigrant background, parental education, and parent’s age when the child was born. Missing information on parental immigrant background was coded as a separate category and included in the analysis. PI = Parental incarceration.

a Before the child turned 12 years old. Missing values were excluded from the analysis (*n* = 212).

In the model stratified by the length of co-residence (Table 2), the associations between PI and violent victimization were stronger among children who had a longer history of co-residence with the incarcerated parent. The HRs for children who co-resided for a longer period ranged from 2.5 to 6.4, and for children who co-resided for a shorter period, from 1.6 to 1.9. Relative risks were the highest at age 0–10 years among those with longer co-residence, and overall, for maternal incarceration. However, absolute risks for violent victimization by 18 years were higher among children who had co-resided less years with the parent. Children with incarcerated mothers were an exception since among them, there was no difference in the absolute risk by the length of co-residence (Supplemental Table 5).

We further analyzed the relationship between the victim and the perpetrator using data from the police register (Table 3). For violent victimization at 0 to 17 years, we did not find notably different proportions of parental violence for children with or without PI. However, being victimized by a non-family member more than 15 years older was more frequent among children with versus without PI (21.2%–27.5% vs. 14.7%–15.1%), and this was especially salient for children with an incarcerated mother. However, victimization by a non-family peer was more frequent among children without versus with PI (57.2%–57.7% vs. 38.0%–48.0%). Among children who were victimized at 0 to 6 years, victimization by parental violence was relatively more frequent among those without PI. Congruently with violent victimization at 0 to 17 years, children aged 0 to 6 years with PI were more likely than those without PI to be victimized by non-family members who were more than 15 years older (36.4% vs. 23.8%).

**Table 3.** Proportions of Familial and Non-Familial Victim–Perpetrator Relationships by Parental Incarceration Among Children Born in Finland in 1987–2003 With Follow-Up for 1996–2020.

Victim 0–17 years	Either parent incarcerated				Paternal incarceration				Maternal incarceration			
	PI (*n* = 3,229)		No PI (*n* = 42,684)		PI (*n* = 2,972)		No PI (*n* = 42,941)		PI (*n* = 495)		No PI (45,418)	
	Proportion %	[95% CI]	Proportion %	[95% CI]	Proportion %	[95% CI]	Proportion %	[95% CI]	Proportion %	[95% CI]	Proportion %	[95% CI]
*Perpetrator*												
Father	12.2	[11.1, 13.4]	11.2	[10.9, 11.5]	12.4	[11.2, 13.6]	11.2	[10.9, 11.5]	10.9	[8.5, 14.0]	11.3	[11.1, 11.6]
Mother	5.5	(4.7, 6.3]	5.4	[5.2, 5.6.]	4.9	[4.2, 5.8]	5.4	[5.2, 5.6]	8.5	[6.3, 11.3]	5.3	[5.1, 5.5]
Sibling/spouse of parent/other from the same apartment	5.6	[4.8, 6.4]	4.3	[4.1, 4.5]	5.3	[4.5, 6.1]	4.3	[4.2, 4.5]	6.9	[4.9, 9.5]	4.4	[4.2, 4.6]
Other, non-family member, age difference max 5 years	47.4	[45.7, 49.1]	57.7	[57.3, 58.2]	48.0	[46.2, 49.7]	57.6	[57.2, 58.1]	38.0	[33.8, 42.3]	57.2	[56.8, 57.7]
Other, non-family member, age difference 6–15 years	8.1	[7.2, 9.1]	6.6	[6.4, 6.9]	8.2	[7.3, 9.3]	6.6	[6.4, 6.9]	8.3	[6.1, 11.1]	6.7	[6.5, 6.9]
Other, non-family member, age difference 15+ years	21.3	[19.9, 22.8]	14.7	[14.4, 15.1]	21.2	[19.8, 22.7]	14.8	[14.5, 15.1]	27.5	[23.7, 31.6]	15.1	[14.7, 15.4]
Victim 0–6 years^a^	Either parent incarcerated											
	PI (*n* = 247)		No PI (*n* = 1,745)									
	Proportion %	[95% CI]	Proportion %	[95% CI]								
*Perpetrator*												
Father	41.3	[35.3, 47.5]	45.0	[42.7, 47.4]								
Mother	16.2	[12.1, 21.3]	23.7	[21.7, 25.7]								
Sibling/spouse of parent/other from the same apartment	4.0	[2.2, 7.4]	4.9	[4.0, 6.0]								
Other, non-family member, Age difference 15+ years	36.4	[30.7, 42.6]	23.8	[21.8, 25.8]								

*Note.* Violent victimization was measured using the police register. PI at 0–17 years. Cases where the offender was unknown were excluded from the analysis (*n* = 1,342). Proportions were estimated for violent victimization events taking into account that there may be multiple victims and offenders within different case numbers. Thus, victims may appear in the data multiple times. If multiple event dates were coded for a case, they were considered as separate violent victimization events. PI = Parental incarceration.

a Categories other, max 5 years, and other, age difference 6–15 years are not shown due to small cell counts (*n* < 10) to ensure data protection.

The findings were supported by sensitivity analyses. First, the results were similar to the main analysis regardless of the data source used to measure violent victimization (Supplemental Table 7). Second, limiting the analysis to children born between 1996 and 2003 caused no major deviation from the results (Supplemental Table 8). Third, the results remained similar when PI was measured before the first violent victimization (Supplemental Table 9). Fourth, the association of PI and childhood violent victimization was similar but slightly lower among the children with no history of parental violent convictions (Supplemental Table 10). Fifth, in the survival models based on the victim–perpetrator analysis, we found that the relative risks for victimization were overall elevated for children with PI regardless of the victim–perpetrator relationship. The highest relative risk was found for being victimized by perpetrators outside the family with over 15 years’ age difference, and lowest for peer victimization (Supplemental Table 11). Finally, we tested whether the associations were different when co-residence was measured before first PI and found no significant differences compared to the main results (Supplemental Table 12).

## Discussion

Using total population data from Finland, we found that the absolute risk of violent victimization was high among children with PI, especially when the incarcerated parent was the mother. One in five of the children with maternal incarceration were victimized before they turned 18, and the corresponding number for children without maternal incarceration was less than 4%. Overall, in line with our hypothesis, exposure to PI was significantly associated with increased risk of violent victimization, and this elevated risk was observed throughout childhood.

Reflecting on criminological theories, this association could be explained by causal effects of parental criminality and PI, manifesting itself, for example, through increased strain, higher exposure to violent individuals, or stigma following parental incarceration. On the other hand, our study cannot rule out the possible role of shared underlying factors giving rise to the intergenerational association. For example, shared genetic factors contribute to risk taking and other behavioral traits, potentially affecting both parental criminal offending and offspring violent victimization (Frisell et al., 2011; Linnér et al., 2021).

In relative terms the associations between PI and violent victimization were stronger in childhood (ages 0–10) in comparison to adolescence (ages 11–17). This is likely due to younger children spending more time within the family environment. Across all analyses, the risks were the highest when the mother was the incarcerated parent. Although previous research has yielded mixed findings (Wildeman et al., 2018), several mechanisms suggest that maternal incarceration may be especially harmful for children. For example, because mothers are less likely to be imprisoned than fathers because of their lower levels of severe criminality, the resulting negative consequences, such as stigmatization or stress, can be more severe. However, incarcerated mothers may also be in many ways different from incarcerated fathers. For example, incarcerated mothers are substantially more likely to have children with incarcerated individuals than vice versa (Nissinen et al., 2024), and incarcerated women in general are found to suffer from poorer health in comparison to incarcerated men (Rautanen et al., 2024). Therefore, due to cumulative disadvantages or unobserved familial and genetic factors, children of incarcerated mothers are a more selected group than children of incarcerated fathers, which is reflected in higher risk of violent victimization in offspring.

We conducted stratified analysis by the length of co-residence and found, congruently with our hypothesis, that relative risks for violent victimization were the highest for children co-residing for longer with their parent. However, upon examining the absolute risks of victimization, we found that among children with either parent or father incarcerated, the absolute risks were higher for children who had co-resided for a shorter period with the parent. Conversely, we found no such differences in the absolute levels of violent victimization in relation to the length of co-residence for mothers. It is possible that children with maternal PI are overall more affected by the factors contributing to the elevated risk of violent victimization. We did not have good data to assess these factors. However, they may be social – for example, closer contact with the parent regardless of living arrangements, or higher exposure to violent individuals or strain caused by incarceration – or again reflect selection, that is, higher risk regardless of the contact. Nonetheless, the higher relative risks among those with longer co-residence were explained by the level of violent victimization in the reference groups: the absolute risk of victimization was notably lower among children without PI with longer as compared to shorter co-residence. Overall, co-residence moderated the associations, however, this was not apparent when the mother was the incarcerated parent.

Research on victimization in early life has mostly focused on maltreatment, caregiver-based victimization and neglect, and peer victimization (Turanovic, 2023). Our results suggest that a notable proportion of violent victimization is perpetrated by an adult outside the family, and among children with PI, this is particularly noticeable for those with maternal incarceration, and in early childhood (0–6 years). This contradicted our hypothesis, as we expected that violence perpetrated by parents would be more pronounced among children with PI. This novel finding highlights the distinct victimization risks faced by children with PI. One possible mechanism explaining this could be that children with incarcerated parents – particularly children with incarcerated mothers – may have increased exposure to other violent adults, such as relatives, parents’ friends and partners, as well as adults in institutional settings, as children with PI are more likely to be taken into care than other children. The likelihood of marriage is lower among incarcerated individuals (Lawrence-Wills, 2004; Shlafer & Poehlmann, 2010), and thus, we were likely unable to identify unofficial parental relationships thoroughly with register-based data. Furthermore, the likelihood of reporting violence committed by someone outside the family might be higher, especially in the institutional settings. Future studies should aim to further clarify the relationship between child victims and their perpetrators, especially among children with PI.

### Limitations

Violent victimization was measured using the medical, causes of death and police registers, which include only the reported violence. As a result, our study may not provide accurate information on associations between PI and less severe, often underreported forms of violent victimization, nor a broader picture of the different forms of violence children with PI might face. It is likely that the absolute level of violent victimization (including broader forms of the measure) is higher than reported in this study. However, presumably the proportion of violent victimization not reported to the police and thus not detected in our study is lower and thus the resulting bias less severe. It is also unclear how this would affect the relative differences in victimization between children with PI and children without PI. Thus, more research on different types and severity of violence among children with PI is needed. Further, PI was measured using imposed prison sentences only, and the category of “no PI” could include parents who were incarcerated as remand prisoners but were not sentenced as prisoners later. Our results thus reflect the associations between violent victimization and PI of parents found guilty in court, not the possible harms of all PI. The variables in the Statistics Finland’s annual data were measured on the last day of each year. In the victim–perpetrator relationship analysis, the relationship between the victims and the perpetrators was therefore defined on a yearly basis, which means that the results may be imprecise. This limitation also applies to the measurement of co-residing: the measurement method does not identify possible periods of living apart/together during the year. These measurement inaccuracies are likely to lead to an underestimation of the effects that we report.

Despite these limitations, using comprehensive longitudinal data, our study was able to refine the association between PI and childhood violent victimization, and to explore the relationship between the perpetrators and the victims with large samples and without participation and self-report biases. Register-based data used in this study are population-representative and include all individuals born in Finland, from various population subgroups, such as individuals with different ethnicity, socioeconomic status, language, sex, gender identity, sexual orientation, or ability. Although the data are less selected, analyses may mask subgroup-specific patterns. Possible heterogeneity of the associations across different subgroups should be explored in future research. In a similar vein, it is also important to note that most children with PI were not victimized in childhood, and mapping of the possible protective factors against violent victimization is also needed. Furthermore, as previous research has demonstrated country-level differences in offspring outcomes of PI (Besemer et al., 2011), the risk of victimization should be examined in other penal and social welfare contexts. Substantial differences exist across countries and regions, for example, in prison systems, and in how selected the group of children with PI is. In Finland, PI is relatively rare, and the results could be different in the context of higher incarceration rate, for example. Similarly, the results might vary across welfare and healthcare regimes. Regardless, the results inform the design of policies aimed at children with PI. Our results suggest that attention to the elevated risk of violent victimization among children with PI is warranted. As PI is concentrated in a highly disadvantaged population, children with PI are likely to need support services, before, during and after PI. Furthermore, PI may indicate problems that extend beyond family life, and this wide-ranging risk of violent victimization needs to be acknowledged when designing effective support measures.

## Conclusions

Violent victimization in childhood and adolescence can have immediate and long-term harmful consequences for health and well-being. Our results suggest that compared to the rest of the population, children with PI are at notably elevated risk of being victims of violence, and this association is especially strong for children with incarcerated mothers. The risk of violent victimization was moderated by the length of co-residence: the associations between PI and violent victimization were stronger among children who had co-resided longer with the parent. However, this moderation effect was not observed for mothers. Furthermore, a substantial part of the violence is conducted by someone outside the family. Interventions should not only focus on child maltreatment by parents but on the risk of violent victimization more broadly.

## Supplemental Material

sj-docx-1-jiv-10.1177_08862605261452168 – Supplemental material for Parental Incarceration and Children’s Risk of Violent Victimization: An Examination of Risks and Perpetrators’ Relationship With the Victim in Finnish Total Population Data Supplemental material, sj-docx-1-jiv-10.1177_08862605261452168 for Parental Incarceration and Children’s Risk of Violent Victimization: An Examination of Risks and Perpetrators’ Relationship With the Victim in Finnish Total Population Data by Ilona Nissinen, Mikko Aaltonen, Joonas Pitkänen, Pekka Martikainen, Karoliina Suonpää and Antti Latvala in Journal of Interpersonal Violence
