# Supplementary material for: Parental Incarceration and Children’s Risk of Violent Victimization: An Examination of Risks and Perpetrators’ Relationship With the Victim in Finnish Total Population Data
Source: J Interpers Violence. 2026 Jun 5;41(17-18):6885–909. doi: 10.1177/08862605261452168 (PMC13447800; doi:10.1177/08862605261452168)
Supplement: Supplementary file 1 [file sj-docx-1-jiv-10.1177_08862605261452168.docx]

Supplementary material for Parental incarceration and children’s risk of violent victimization: An examination of risks and perpetrators’ relationship with the victim in Finnish total population data
**Supplementary Table 1.** ICD codes to identify violent victimization and violent deaths in the medical and causes of death registers.

|  | **ICD-10-FI (1996–2020)** |
| --- | --- |
|  |  |
| Violent victimization/death | **X85–X99; Y00–Y04, Y06–Y09; Y87.1** |

**Supplementary Table 2.** Types of violent crime in the police register data.

| **Violent crime type** |  | **Criminal offence under Finnish criminal law** |
| --- | --- | --- |
|  |  |  |
| **Petty assault** |  | Petty assault |
| **Assault** |  | Assault |
|  |  | Attempted assault |
| **Aggravated assault** |  | Aggravated assault |
|  |  | Attempted aggravated assault |
|  |  | Aggravated assault committed with a terrorist intent |
|  |  | Attempted aggravated assault committed with a terrorist intent |
|  |  | Attempted manslaughter |
|  |  | Attempted murder |
|  |  | Attempted killing |

**Supplementary Table 3.** Parent’s level of education on the 7-category classification.

|  | **Either parent** | | **Fathers** | | **Mothers** | |
| --- | --- | --- | --- | --- | --- | --- |
|  | PI 0–17 yrs (n=18,707) | No PI 0–17 yrs (n=998,365) | PI 0–17 yrs (n=17,600) | No PI 0–17 yrs (n=999,472) | PI 0–17 yrs  (n=1,859) | No PI 0–17 yrs  (n= 1,015,213) |
|  | % | % | % | % | % | % |
| Comprehensive school or lower | 19.5 | 3.2 | 52.4 | 16.7 | 61.4 | 9.3 |
| Upper secondary level | 57.6 | 35.6 | 41.1 | 45.0 | 31.3 | 40.9 |
| Post-secondary non-tertiary education | 2.4 | 2.5 | 0.6 | 2.0 | 0.9 | 1.9 |
| Short-cycle tertiary education | 9.6 | 21.8 | 3.2 | 14.0 | 3.8 | 21.2 |
| Bachelor’s or equivalent level | 7.0 | 15.5 | 1.6 | 9.4 | 1.7 | 11.7 |
| Master’s or equivalent level | 3.6 | 18.6 | 1.0 | 11.2 | 1.0 | 13.7 |
| Doctoral or equivalent level | 0.4 | 2.8 | 0.1 | 1.8 | 0.0 | 1.3 |

PI=Parental incarceration

**Supplementary Table 4.** Timing of violent victimization in relation to the parent's first period of incarceration among children born in Finland in 1987-2003 with follow-up for 1996-2020.

|  | **Either parent incarcerated** | **Father incarcerated** | **Mother incarcerated** |
| --- | --- | --- | --- |
| First violent victimization event happened before the first parental incarceration | 336 (14.1 %) | 284 (13.0 %) | 83 (22.7 %) |
| First violent victimization event happened on the same year or after the first parental incarceration | 2,052 (85.9 %) | 1,903 (87.0 %) | 282 (77.3 %) |

**Supplementary Table 5.** Cumulative incidence (95% CI) of violent victimization in the offspring by age 18 by parental incarceration and the length of co-residence among children born in Finland in 1987-2003 with follow-up for 1996-2020.

|  | **Either parent incarcerated** | | **Paternal incarceration** | | **Maternal incarceration** | |
| --- | --- | --- | --- | --- | --- | --- |
| **Total population** | | | | | | |
|  | PI (n=18,707) | No PI (n=998,365) | PI (n=17,600) | No PI (n=999,472) | PI (n=1,859) | No PI (n= 1,015,213) |
| **Proportion with violent victimization** | 12.8 (12.4–13.3) | 3.4 (3.4–3.4) | 12.5 (12.0–13.0) | 3.4 (3.4–3.5) | 20.0 (18.1–21.9) | 3.5 (3.5–3.6) |
| **Co–residence for maximum of six years with the parent^a^** | | | | | | |
|  | **Either parent incarcerated** | | **Paternal incarceration** | | **Maternal incarceration** | |
|  | PI (n=2,014) | No PI (n=11,672) | PI (n=13,218) | No PI (n=168,799) | PI (n=907) | No PI (n=26,070) |
| **Proportion with violent victimization** | 16.2 (14.6–18.0) | 7.3 (6.7–8.1) | 13.1 (12.5–13.7) | 7.0 (6.9–7.1) | 18.8 (16.3–21.5) | 8.4 (8.0–8.8) |
| **Co–residence for over six years with the parent^a^** | | | | | | |
|  | **Either parent incarcerated** | | **Paternal incarceration** | | **Maternal incarceration** | |
|  | PI (n=16,693) | No PI (n=986,481) | PI (n=4,382) | No PI (n=830,461) | PI (n=952) | No PI (n=988,931) |
| **Proportion with violent victimization** | 12.5 (12.0–13.0) | 3.4 (3.3–3.4) | 10.9 (10.0–11.9) | 2.7 (2.7–2.8) | 21.1 (18.5–23.9) | 3.5 (3.4–3.5) |

Violent victimization was measured using medical, causes of death, and police register.
PI=Parental incarceration
PI at 0–17 yrs.
^a^ Missing values were excluded from the analysis (n=212).

**Supplementary Table 6.** Descriptive statistics on police data for children born in Finland in 1987-2003 with follow-up for 1996-2020.

| **Number of unique victims in the data** | **34,384** |
| --- | --- |
| Victim linked to one case only | 29,665 |
| Victim linked to multiple cases | 4,719 |
| **Number of unique offenders in the data** | **34,404** |
| Offender linked to one case only | 29,268 |
| Offender linked to multiple cases | 5,136 |
| **Number of cases where the offender is unknown** | **1,342** |
| **Type of violent victimization %** |  |
| Petty assault | 27.5 |
| Assault | 69.5 |
| Aggravated assault^a^ | 3.1 |

Victim 0–17 years old.
 ^a^Aggravated assaults included attempted homicides.

**Supplementary Table 7.** Associations of parental incarceration with offspring violent victimization in childhood among children born in Finland in 1987-2003 with follow-up for 1996-2020. Reference categories: no parental / paternal / maternal PI. Violent victimization measured using the medical and police registers separately.

|  | **Either parent incarcerated** | | **Paternal Incarceration** | | **Maternal Incarceration** | |
| --- | --- | --- | --- | --- | --- | --- |
| **Medical register** |  |  |  |  |  |  |
|  | HR | (95 % CI) | HR | (95 % CI) | HR | (95 % CI) |
| **Violent victimization** |  |  |  |  |  |  |
| at 0–17 yrs | 2.8 | (2.5–3.2) | 2.8 | (2.4–3.2) | 3.8 | (2.8–5.1) |
| **Police register** |  |  |  |  |  |  |
|  | HR | (95 % CI) | HR | (95 % CI) | HR | (95 % CI) |
| **Violent victimization** |  |  |  |  |  |  |
| at 0–17 yrs | 2.6 | (2.5–2.7) | 2.7 | (2.5–2.8) | 3.1 | (2.8–3.5) |

Medical register includes data from the medical and causes of death registers.
PI=Parental incarceration
All models were adjusted for child’s sex, birth year, parental immigrant background, parental education, and parent’s age when the child was born. Missing information on parental immigrant background was coded as a separate category and included in the analysis.

**Supplementary Table 8.** Associations of parental incarceration with offspring violent victimization in childhood among children born in Finland in 1996-2003 with follow-up for 1996-2020. Reference categories: no parental / paternal / maternal PI.

|  | **Either parent incarcerated** | | **Paternal incarceration** | | **Maternal incarceration** | |
| --- | --- | --- | --- | --- | --- | --- |
|  | HR | (95 % CI) | HR | (95 % CI) | HR | (95 % CI) |
| **Violent victimization** |  |  |  |  |  |  |
| at 0–17 yrs | 2.7 | (2.6–2.9) | 2.7 | (2.5–2.9) | 3.4 | (2.9–4.0) |
| at 0–10 yrs | 3.5 | (3.1–3.9) | 3.6 | (3.2–4.1) | 4.1 | (3.2–5.3) |
| at 11–17 yrs | 2.5 | (2.3–2.7) | 2.4 | (2.2–2.6) | 3.1 | (2.6–3.7) |

Violent victimization was measured using data from the medical, causes of death and police registers.
PI=Parental incarceration
PI at 0–17 yrs.
All models were adjusted for child’s sex, birth year, parental immigrant background, parental education, and parent’s age when the child was born. Missing information on parental immigrant background was coded as a separate category and included in the analysis.

**Supplementary Table 9.** Associations of parental incarceration experienced at 0–5 yrs/0–11 yrs with offspring violent victimization at 6–17 yrs/12–17 yrs among children born in Finland in 1987-2003 with follow-up for 1996-2020. Reference categories: no parental / paternal / maternal PI at 0–5 yrs/0–11 yrs.

|  | **Either parent incarcerated** | | **Paternal Incarceration** | | **Maternal Incarceration** | |
| --- | --- | --- | --- | --- | --- | --- |
|  | HR | (95 % CI) | HR | (95 % CI) | HR | (95 % CI) |
| **Violent victimization** |  |  |  |  |  |  |
| at 6–17 yrs^a^ | 2.5 | (2.4–2.6) | 2.6 | (2.5–2.8) | 2.9 | (2.4–3.6) |
| at 12–17 yrs^b^ | 2.4 | (2.3–2.5) | 2.5 | (2.3–2.6) | 2.7 | (2.3–3.2) |

Violent victimization was measured using the medical, causes of death, and policer registers.
PI=Parental incarceration

^a^PI at 0–5 yrs.

^b^PI at 0–11 yrs.
All models were adjusted for child’s sex, birth year, parental immigrant background, parental education, and parent’s age when the child was born. Missing information on parental immigrant background was coded as a separate category and included in the analysis.

**Supplementary Table 10.** Associations of parental incarceration with offspring violent victimization among children with no history of parental violent convictions among children born in Finland in 1987-2003 with follow-up for 1996-2020. Reference categories: no parental / paternal / maternal PI at 0–17yrs.

|  | **Either parent incarcerated (n=7,252)** | | **Paternal Incarceration (n=7,292)** | | **Maternal Incarceration (n=842)** | |
| --- | --- | --- | --- | --- | --- | --- |
|  | HR | (95 % CI) | HR | (95 % CI) | HR | (95 % CI) |
| **Violent victimization** |  |  |  |  |  |  |
| at 0–17 yrs | 2.4 | (2.2–2.6) | 2.4 | (2.3–2.6) | 2.7 | (2.3–3.3) |

Violent victimization was measured using the medical, causes of death, and policer registers.
PI=Parental incarceration

All models were adjusted for child’s sex, birth year, parental immigrant background, parental education, and parent’s age when the child was born. Missing information on parental immigrant background was coded as a separate category and included in the analysis.

**Supplementary Table 11.** Associations of parental incarceration with offspring violent victimization by perpetrator among children born in Finland in 1987-2003 with follow-up for 1996-2020. Reference category: no PI at 0–17yrs.

|  | **Either parent incarcerated** | |
| --- | --- | --- |
|  |  |  |
| *Perpetrator* | HR | 95 % CI |
| Father | 3.0 | (2.7–3.4) |
| Mother | 2.5 | (2.1–2.9) |
| Sibling/spouse of parent/other from the same apartment | 2.8 | (2.4–3.3) |
| Other, non-family member, age difference max 5 yrs | 2.2 | (2.1–2.4) |
| Other, non-family member, age difference 6–15 yrs | 2.9 | (2.5–3.3) |
| Other, non-family member, age difference 15+ yrs | 3.3 | (3.0–3.6) |

PI=Parental incarceration

Victim 0–17 yrs.
PI at 0–17 yrs.
Cases where the offender was unknown were excluded from the analysis (n=1,342).
All models were adjusted for child’s sex, birth year, maternal immigrant background, maternal education, and mother´s age when the child was born. Missing information on parental immigrant background was coded as a separate category and included in the analysis.

**Supplementary Table 12.** Associations of parental incarceration with offspring violent victimization stratified by co-residence at 0–5/6–11 yrs among children born in Finland in 1987-2003 with follow-up for 1996-2020. Reference categories: no parental / paternal / maternal PI at 6–17/12-17 yrs.

|  | **Either parent incarcerated** | | | | **Paternal Incarceration** | | | | **Maternal Incarceration** | | | |
| --- | --- | --- | --- | --- | --- | --- | --- | --- | --- | --- | --- | --- |
|  | **A maximum of three years lived with parent^a^** | | **Over three years lived with parent^a^** | | **A maximum of three years lived with parent^a^** | | **Over three years lived with parent^a^** | | **A maximum of three years lived with parent^a^** | | **Over three years lived with parent^a^** | |
|  | HR | (95 % CI) | HR | (95 % CI) | HR | (95 % CI) | HR | (95 % CI) | HR | (95 % CI) | HR | (95 % CI) |
| **Violent victimization** |  |  |  |  |  |  |  |  |  |  |  |  |
| at 0–17 | 1.6 | (1.1–2.3) | 2.5 | (2.4–2.7) | 1.4 | (1.2–1.5) | 2.9 | (2.6–3.2) | 1.4 | (1.0–1.9) | 3.6 | (3.2–4.2) |
|  | **A maximum of six years lived with parent^b^** | | **Over six years lived with parent^b^** | | **A maximum of six years lived with parent^b^** | | **Over six years lived with parent^b^** | | **A maximum of six years lived with parent^b^** | | **Over six years lived with parent^b^** | |
|  | HR | (95 % CI) | HR | (95 % CI) | HR | (95 % CI) | HR | (95 % CI) | HR | (95 % CI) | HR | (95 % CI) |
| **Violent victimization** |  |  |  |  |  |  |  |  |  |  |  |  |
| at 0–17 | 1.9 | (1.2–2.8) | 2.7 | (2.4–3.0) | 1.4 | (1.2–1.6) | 3.5 | (3.0–4.1) | 2.1 | (1.5–3.0) | 3.8 | (3.1–4.8) |

Violent victimization was measured using the medical, causes of death, and policer registers.
PI=Parental incarceration
^a^PI at 6–17 yrs, no PI at 0-5 yrs.
^b^PI at 12–17 yrs, no PI at 0-11 yrs.
All models were adjusted for child’s sex, birth year, parental immigrant background, parental education, and parent’s age when the child was born. Missing information on parental immigrant background was coded as a separate category and included in the analysis.
